# Inhibition of GSK_3β by Iridoid Glycosides of Snowberry (*Symphoricarpos albus*) Effective in the Treatment of Alzheimer’s Disease Using Computational Drug Design Methods

**DOI:** 10.3389/fchem.2021.709932

**Published:** 2021-10-07

**Authors:** Marzieh Eskandarzadeh, Parastou Kordestani-Moghadam, Saeed Pourmand, Javad Khalili Fard, Bijan Almassian, Sajjad Gharaghani

**Affiliations:** ^1^ Research Committee of Faculty of Pharmacy, Lorestan University of Medical Science, Khorramabad, Iran; ^2^ Social Determinants of Health Research Center, Lorestan University of Medical Sciences, Khorramabad, Iran; ^3^ Department of Chemical Engineering, Faculty of Chemical and Petroleum Engineering, University of Tabriz, Tabriz, Iran; ^4^ Razi Herbal Medicines Research Center, Lorestan University of Medical Sciences, Khorramabad, Iran; ^5^ Department of Pharmacology and Toxicology, Faculty of Pharmacy, Tabriz University of Medical Sciences, Tabriz, Iran; ^6^ CaroGen Corporation, Farmington, CT, United States; ^7^ Laboratory of Bioinformatics and Drug Design, Institute of Biochemistry and Biophysics, University of Tehran, Tehran, Iran

**Keywords:** loganin, Alzheimer’s disease, GSK_3 beta, docking, *Symphoricarpos*

## Abstract

The inhibition of glycogen synthase kinase-3β (GSK-3β) activity prevents tau hyperphosphorylation and binds it to the microtubule network. Therefore, a GSK-3β inhibitor may be a recommended drug for Alzheimer’s treatment. In silico methods are currently considered as one of the fastest and most cost-effective available alternatives for drug/design discovery in the field of treatment. In this study, computational drug design was conducted to introduce compounds that play an effective role in inhibiting the GSK-3β enzyme by molecular docking and molecular dynamics simulation. The iridoid glycosides of the common snowberry (*Symphoricarpos albus*), including loganin, secologanin, and loganetin, are compounds that have an effect on improving memory and cognitive impairment and the results of which on Alzheimer’s have been studied as well. In this study, in the molecular docking phase, loganin was considered a more potent inhibitor of this protein by establishing a hydrogen bond with the ATP-binding site of GSK-3β protein and the most negative binding energy to secologanin and loganetin. Moreover, by molecular dynamics simulation of these ligands and GSK-3β protein, all structures were found to be stable during the simulation. In addition, the protein structure represented no change and remained stable by binding ligands to GSK-3β protein. Furthermore, loganin and loganetin have higher binding free energy than secologanin; thus, these compounds could effectively bind to the active site of GSK-3β protein. Hence, loganin and loganetin as iridoid glycosides can be effective in Alzheimer’s prevention and treatment, and thus, further *in vitro* and *in vivo* studies can focus on these iridoid glycosides as an alternative treatment.

## Introduction

Alzheimer’s disease (AD) is the most prevalent form of dementia (Lin, Jones et al., 2020). This health problem results in malignant neurological disorder along with cognitive, functional, and behavioral changes progressively and irreversibly. According to statistics, by 2050, this age-associated disease will affect 1 out of 85 people globally (Brookmeyer et al., 2007), posing a social and economic burden on the future community (Association 2019).

AD has a multifactorial etiology, with the most prominent ones associated with extracellular deposition of amyloid-β (Aβ) plaques, aggregation of tau protein, inflammation, oxidative stress, and declined levels of acetylcholine (De Strooper and Karran 2016; Hall et al., 2019). Nowadays, some evidence suggests that Aβ deposition is related to aging abnormalities. Also, tau (*τ*) protein is probably a higher target than Aβ because several therapeutic techniques concerning Aβ have not been highly effective in the disease treatment (Kametani and Hasegawa 2018). Tau protein is a member of the microtubule-related protein family that promotes microtubule stability and cytoskeleton formation (Kolarova et al., 2012). Intracellular tau protein aggregation in nerve fiber nodes is a neuropathological feature of AD. In the brain of people with AD, tau protein undergoes hyperphosphorylation because of some factors such as, formation of Aβ and environmental factors etc (Gong and Iqbal 2008). Thus, it is separated from microtubules (Mandelkow et al., 2007). Consequently, it results in neurofibrillary tangles (NFTs), ultimately leading to synapse loss and nerve cell destruction (Lauretti et al., 2020). Different kinases and phosphatases regulate the degree of phosphorylation of tau protein (Kolarova et al., 2012). In people with AD, tau protein is hyperphosphorylated, and it causes tauopathy due to degranulation and imbalance between them (Martin et al., 2013). One of the major kinases involved in tau protein phosphorylation is the glycogen synthase kinase_3 (GSK-3) enzyme (Jouanne et al., 2017). This serine–threonine kinase has two isoforms of alpha and beta (GSK3-α and GSK3-β) (Bagyinszky et al., 2014), which are encoded by two different genes (Lee et al., 2006).

In the central nervous system of people with AD, the GSK3-β enzyme is hyperactive, and evidence supports its role in AD pathology (Llorens-Marítin et al., 2014). GSK3-β phosphorylates many tau protein sites among people with AD and results in hyperphosphorylation of tau protein. Moreover, GSK3-β hyperactivity has been associated with impaired neurogenesis, microglia activation, amyloid-beta (Aβ) deposition, and inhibition of long-term potentiation (LTP) hippocampus and memory impairment (Llorens-Marítin et al., 2014).

This protein has two lobes, i.e., n-terminal and c-terminal, with residues 25 to 134 located in the N-terminal region and 35 to 380 in the c-lobe. Furthermore, these two lobes are held together by the hinge area such that residues 133 to 139 are considered a part of this area. Activation LOOP (A-Loop) is one of the most important loops of this enzyme where residues of A-LOOP are phosphorylated and have a key role in enzyme regulation. Among the residues of this loop, the DFG motif (ASP200-GLY202) is vital in enzyme regulation (ter Haar, Coll et al., 2001; Elangovan, Dhanabalan et al., 2020).

GSK3-β maintains an ATP-binding site between the two lobes. An important region of the ATP-binding site is the hinge region containing the residues Asp133, TYR134, and Val135. Some studies revealed that VAL135 and AP133 are very important residues for the binding of small molecules to the ATP-binding site (Gyurak et al., 2012; Pandey and DeGrado 2016; Davies 2019).

Hence, given the various roles of GSK3-β in AD pathology, the therapeutic potentials for its inhibition have been extensively examined in different studies.

Currently, there are no definitive treatments for AD (Eftekharzadeh et al., 2018), and the FDA-approved medicines for the disease are only for symptomatic treatment to improve behavioral changes and delay performance decline. These medicines include acetylcholinesterase inhibitors (e.g., donepezil, rivastigmine, and galantamine) and the N-methyl-D-aspartate receptor (NMDAR) (memantine). To date, several attempts have been made to find new inhibitors for AD, such as finding GSK3-β inhibitors.

The common snowberry plant (with the scientific name *Symphoricarpos albus*) has a fruit rich in phenolic acids, carbohydrates, and iridoids. The most important iridoid glycosides of this plant are loganin, loganetin, and secologanin (Gilbert 1995; Makarevich et al., 2009). Many biological activities like antioxidant, anti-inflammatory, anticancer, antidiabetic, antimicrobial, antiviral, anti-prosthetic, and neuroprotective activities have been reported for iridoid glycosides (Dinda et al., 2007; Dinda et al., 2019). In this respect, a study showed that iridoid glycosides effectively improve memory and cause nerve cell survival by increasing the expression of synaptophysin and neurotrophic factors in Alzheimer’s mice with cholinergic defects. Additionally, these compounds enhance cognitive impairment and inhibit hyperphosphorylation of tau protein in the hippocampus and striatum of SAMP8 mice (Ma et al., 2016).

Another study indicated that iridoid glycosides could increase pp2a activity, decrease GSK3-β activity, and finally inhibit tau hyperphosphorylation. Therefore, these treatment options can effectively improve cognitive and behavioral disorders in AD patients (Wang et al., 2020; Zhang et al., 2020). In this respect, we have used computational drug design methods, which are one of the fastest and most cost-effective ways available for drug design and finding new inhibitors of the GSK3- *β* enzyme. Moreover, we examined the inhibitory potential of the GSK3-β enzyme by loganin, loganetin, and secologanin with the help of molecular docking methods and molecular dynamic (MD) simulation.

## Materials and Methods

### Molecular Docking

#### Validation

Before molecular docking, we need to validate the software processing. Validation is of vital significance as it will eliminate false results. Since GSK3-β inhibition can be a treatment for AD, many inhibitors have been examined; many of them are competitive inhibitors with the ATP site and target its binding site in GSK3-β. The co-crystal structures of these inhibitors can be obtained through the Protein Data Bank (PDB) site. Codes related to the GSK3-β protein as a complex with the inhibitor are 1Q41 (Bertrand et al., 2003), 1Q3D (Bertrand et al., 2003), 1Q4L (Bertrand et al., 2003), 2OW3 (Zhang et al., 2007), 1R0E (Allard. 2004), 1Q3W (Bertrand et al., 2003), 3GB2 (Saitoh et al., 2009), 3F88 (Saitoh et al., 2009), 3F7Z (Saitoh et al., 2009), 3I4B (Aronov, Tang et al., 2009), 1UV5 (Meijer et al., 2003), 3Q3B (Coffman et al., 2011), 3L1S (Arnost et al., 2010), 1Q5K (Bhat et al., 2003), and 2O5K (Shin et al., 2007).

After downloading the PDB file corresponding to each of these codes, the structure of the GSK3-β protein and the cognate ligand of each code were prepared and optimized in ViewerLite 5.0 (Lite, 1998) software. All water molecules and nonpolar hydrogen of protein and nonpolar hydrogen of inhibitors were removed. In the next step, we performed redocking for each complex using both AutoDock4.2 (Forli et al., 2012) and AutoDock Vina (Trott and Olson 2010) software. We have used the two software to select the most appropriate software during the validation phase. Moreover, 100 runs were considered for each complex. Afterward, the best ligand conformation with the most negative ΔG and the lowest root mean square deviation (RMSD) was considered for each complex. Eventually, using Visual Molecular Dynamics (VMD**)** 1.9.3(http://www.ks.uiuc.edu) software, we obtained the RMSD between the selected conformations for the ligand and the ligand in the obtained crystalline structure from the PDB site.

In the next step, the obtained RMSDs of redock using two software were compared to each other for choosing the best software. Between these two tools, whichever showed the lower RMSD than the other, we chose it for loganin, secologanin, and loganetin docking with GSK3-β because it means that it can better predict the active site of the enzyme than the other.

Additionally, after selecting the software, the code with the lowest RMSD was used as the selected code for protein–ligand docking.

### Docking Loganin, Secologanin, and Loganetin With GSK3-β Protein

#### Ligand Structure Preparation

First, the two-dimensional structure of each ligand was prepared in the ChemDraw Ultra 2d 8.0 (Mendelsohn 2004) tool and then was transmitted into ChemDraw Ultra 3d 8-0 (Mendelsohn 2004) software for preparing the three-dimensional structure. Then, the structures of ligands were subjected to energy minimization with the default of the MOPAC program in ChemDraw Ultra 3d 8.0 and then were saved as in the format of **.**mol. So, we converted them to the PDB format by ViewerLite 5.0 and used them as inputs of selected software for molecular docking.

The Gasteiger–Marsili procedure was used for calculating the partial charges of atoms (Shahlaei et al., 2011). Nonpolar hydrogens were deleted and then rotatable bonds were determined. All rotatable bonds of ligands were assumed to be flexible.

### Protein Structure Preparation

We used the protein structure related to a selected complex in the validation step that it had lowest RMSD. And then, all water molecules and co-crystallized ligands were removed by the ViewerLite 5.0 tool, and just chain A of the protein was considered for molecular docking. Afterward, all hydrogens were added, and nonpolar hydrogens were removed by the selected tool. Then, Kolman charges were determined for the protein.

### Docking Procedure

Molecular docking was performed by selected software in the validation stage. We used the Lamarckian genetic algorithm (LGA) method in AuotoDock4.2 based on the previous studies because they have revealed that other approaches (simulated annealing and genetic algorithm) are less effective than LGA (Morris et al., 1998). A total of 100 independent runs were considered for each ligand.

The dimensions of the center grid box for loganin, loganetin, and secologanin were considered 94.63 A° × 68.1680 A° × 9.7880 A°, and the software was allowed to search the entire volume of the protein. After finishing the docking, the two-dimensional figures of the results (e.g., the binding site and interactions between GSK3-β and inhibitors) were exhibited using LigPlot^+^ (Laskowski and Swindells 2011) software.

### Molecular Dynamic Simulation

We carried out MD simulation in two steps. First, the structure of GSK3-β from a selected complex in the validation step was simulated in a water box. Second, to investigate the effects of ligand binding on GSK3-β conformation, the lowest binding energy conformation from the docking step of ligands was imported into the MD simulation.

For simulation, we used GROMAX 2019.1 (https://doi.org/10.5281/zenodo.2564761) software. At first, the protein was modified using the Swiss_PDB viewer (SPDBV) (Kaplan and Littlejohn 2001) program since the protein had the missing atom. Then, all the heteroatoms and water molecules were removed from the protein. In the next stage, topology and coordinate files were prepared. CHARMM27 was used as a force field, with the intermolecular (nonbonded) potential demonstration as a sum of Lennard–Jones (LJ) force and pairwise Coulomb interaction, and the long-range electrostatic force was defined by the particle mesh Ewald (PME) method. The velocity Verlet algorithm was applied for the numerical intermixture (Gharaghani et al., 2013).

Under periodic boundary conditions, the system was placed in a cubic water box of extended simple point-charge (SPC) water molecules with dimensions of 9.0465 × 9.06465 × 9.06465 nm^3^ with 1 nm away from the wall on each side.

Moreover, the system has to be examined for electrostatic neutrality, and anions or cations should be added if necessary. Here, 6 chloride ions were added to neutralize the system, and the entire system was included 5,579 atoms of GSK3-β, 6 Clˉ, and 22,560 solvent atoms. In the next stage, the energy was minimized in 50,000 steps for 2 fs. Then, the system was equilibrated at a constant temperature (NVT) of 300 k using the Berendsen thermostat. The cutoff radius was considered as 1.2 nm. Then, the system was balanced in the same way at a constant pressure (NVT) of 1 bar.

The goal of balancing the system at constant pressure and temperature for solvent molecules was to reach their best arrangement around the solute. Ultimately, the system was placed in the main run of the simulation for 100 ns at 300 K temperature and 1 bar pressure. To simulate the GSK3-β–ligand complex, first, the ligand-related topology file was generated using the SWISSPARAM server for the CHARMM27 force field. Afterward, the topology parameters and ligand coordinates were added to the topology parameters and coordinates of GSK3-β. Molecular dynamic simulation of the protein–ligand complex was performed similar to protein simulation for 100 ns. Ultimately, all the analyses related to the simulation were carried out in GROMACS 2019.1 software. These analyses include the evaluation of intermolecular hydrogen bonds, RMSD, Rg, RMSF, and free binding energy using the MM–GBSA method. The MD simulation was carried out on Ubuntu 18.04 Linux on an Intel Core 12 Quad 6800K 3.6 GHz, Gpu = gtx 1080ti NVIDIA, and 16 GB RAM.

### Binding Free Energy Calculation

The binding free energy is a critical step of an in silico drug design approach which determines the binding affinity of inhibitors to the receptor. The binding free energy was calculated for the ligand receptor of loganin, loganetin, and secologanin complexes using MM/PBSA which defines the binding free energy of the protein and ligand as

$$\Delta G_{\mathrm{binding}}=\Delta G_{\mathrm{complex}}-\left(\Delta G_{\mathrm{protein}}+\Delta G_{\mathrm{ligand}}\right)$$ (1)

Here, ΔG_complex_ demonstrates the total MMPBSA energy of the protein–ligand complex; ΔG_protein_, and ΔG_ligand_ are solution free energies of the individual protein and ligand, respectively. The free energy of the individual existence can be expressed as follows:

$$\Delta G=E_{\mathrm{MM}}+G_{\mathrm{solvation}}-\mathrm{TS}$$ (2)

E_MM_ shows the average molecular mechanical’s potential energy in the vacuum; G_solvation_ defines the free energy of solvation. T and S explain the temperature and entropy, respectively, and together TS exhibits the entropic contribution to the free energy in vacuum. In addition, the E_MM_ contains both bonded and nonbonded interactions of the molecules, including the bond angle, torsion, and electrostatic (E_elec_) and van der Waals (E_vdw_) interactions. Last, the free energy of solvation and G_solvation_ include both electrostatic and nonelectrostatic (G_polar_ and G_nonpolar_) components. The binding free energies of the loganin_GSK3-β, loganetin_GSK3-β, and secologanin_GSK3-β complexes were calculated for 200 snapshots obtained from the last 20 ns of the trajectories (Padhi et al., 2021).

### Drug Likeness Prediction and Toxicity

The OSIRIS property explorer (http://www.organic-chemistry.org/prog/peo/) was used to investigate the likeness of drugs. Pharmacokinetic properties like Log S calculation, TPSA, Clog P calculation, molecular mass, and toxicities like mutagenicity, tumorigenicity, irritation force, and hazard of the reproductive force of three compounds have been established.

## Results and Discussion

### Molecular Docking

In the molecular docking stage, it was seen that the obtained RMSD by AutoDock4.2 in most codes is less than the obtained results by AutoDock Vina software (Table 1). Since the AutoDock4.2 software can accurately predict the active site of GSK3-β protein, it was selected to dock our ligands. Moreover, according to the RMSD results of all codes by this software, the code 1UV5 with RMSD = 0.6679 had the lowest RMSD compared to other codes. 1UV5 contains the 6-Bromine dirubio-3-oxime inhibitor (BRW1383), which inhibits GSK3-β protein competitively with the ATP active site.

**TABLE 1:** Result of redock by AutoDock4.2 and AutoDock Vina software.

Code	RMSD of AutoDock4.2	RMSD of AutoDock Vina
1Q4L	2.4015	2.9621
1Q3D	3.011	2.4863
1Q41	6.2644	6.816
1Q3W	1.2191	5.0456
1R0E	4.27	6.02693
2OW3	4.0532	3.9890
1UV5	0.6679	0.5720
3I4B	7.3559	7.1482
3F7Z	4.7133	5.2366
3F88	5.916	5.7504
3GB2	3.3699	1.8433
1Q5K	6.7193	8.9578
2O5K	6.3006	10.6104
3L1S	4.115	5.2689
3Q3B	6.8446	6.77

**FIGURE 1:**
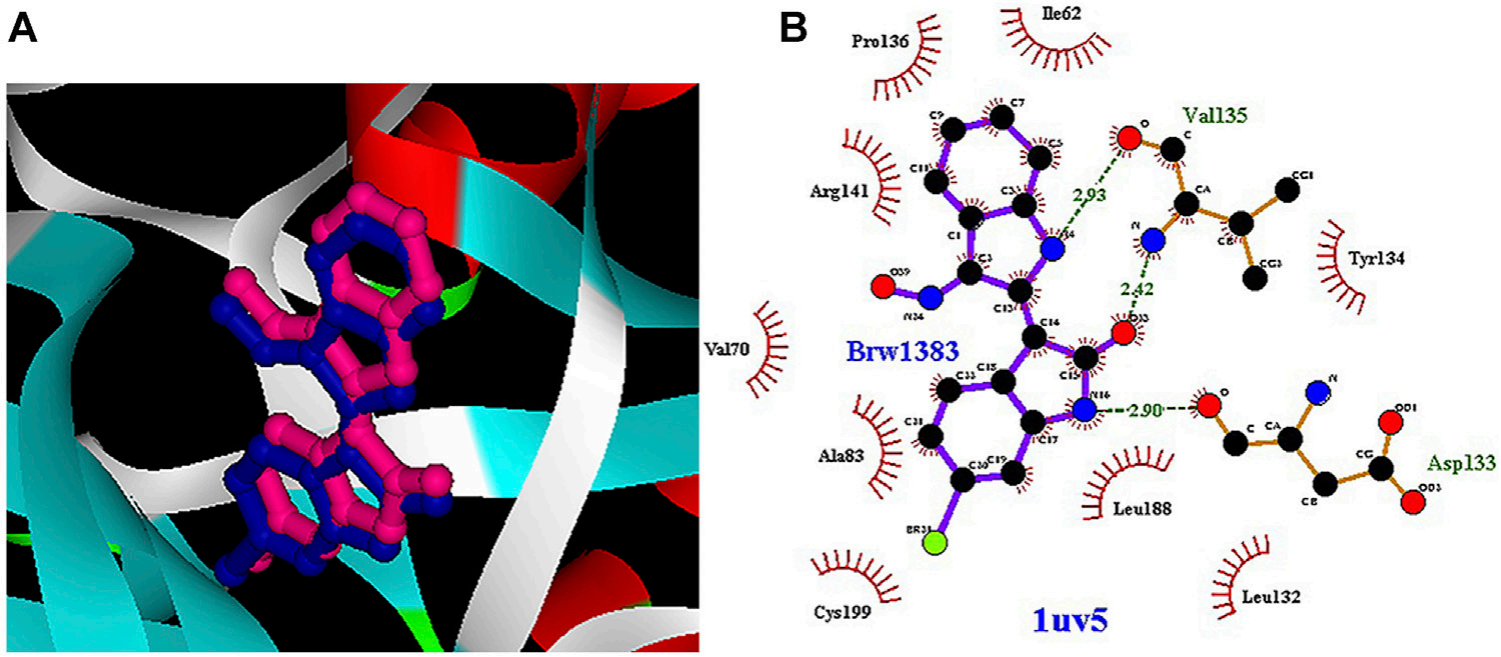
**(A)** Interacted BRW1383 (pink) and BRW1383 from X-ray crystallography (blue). **(B)** Interactions of BRW1383 with GSK3_β protein obtained from X-ray crystallography.

Among the most important amino acids with a crucial role in regulating this kinase, one can name ASP133, ASP200, and VAL135. As shown in Figure 1A, interacted BRW1383 in AutoDock 4.2 software with a −9.56 Kcal/mol binding energy could be matched on the BRW1383 from X-ray crystallography well and could establish the same bonds as those reported in the X-ray crystallographic structure (Figure 1B).

**FIGURE 2:**
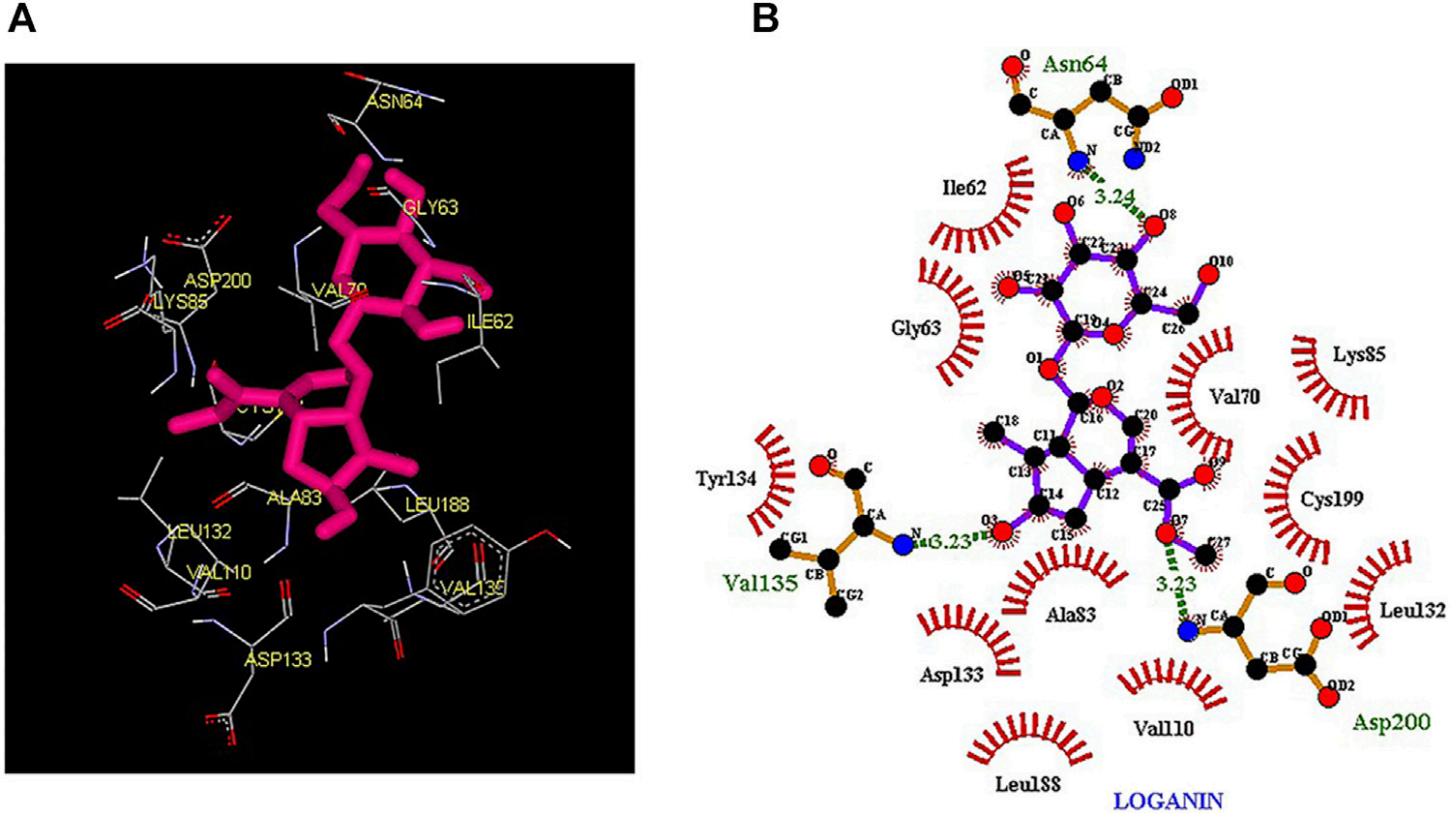
**(A)** Three-dimensional view of loganin at the GSK_3β protein binding site. **(B)** Interactions of loganin with amino acids of the GSK3_β protein binding site.

**FIGURE 3:**
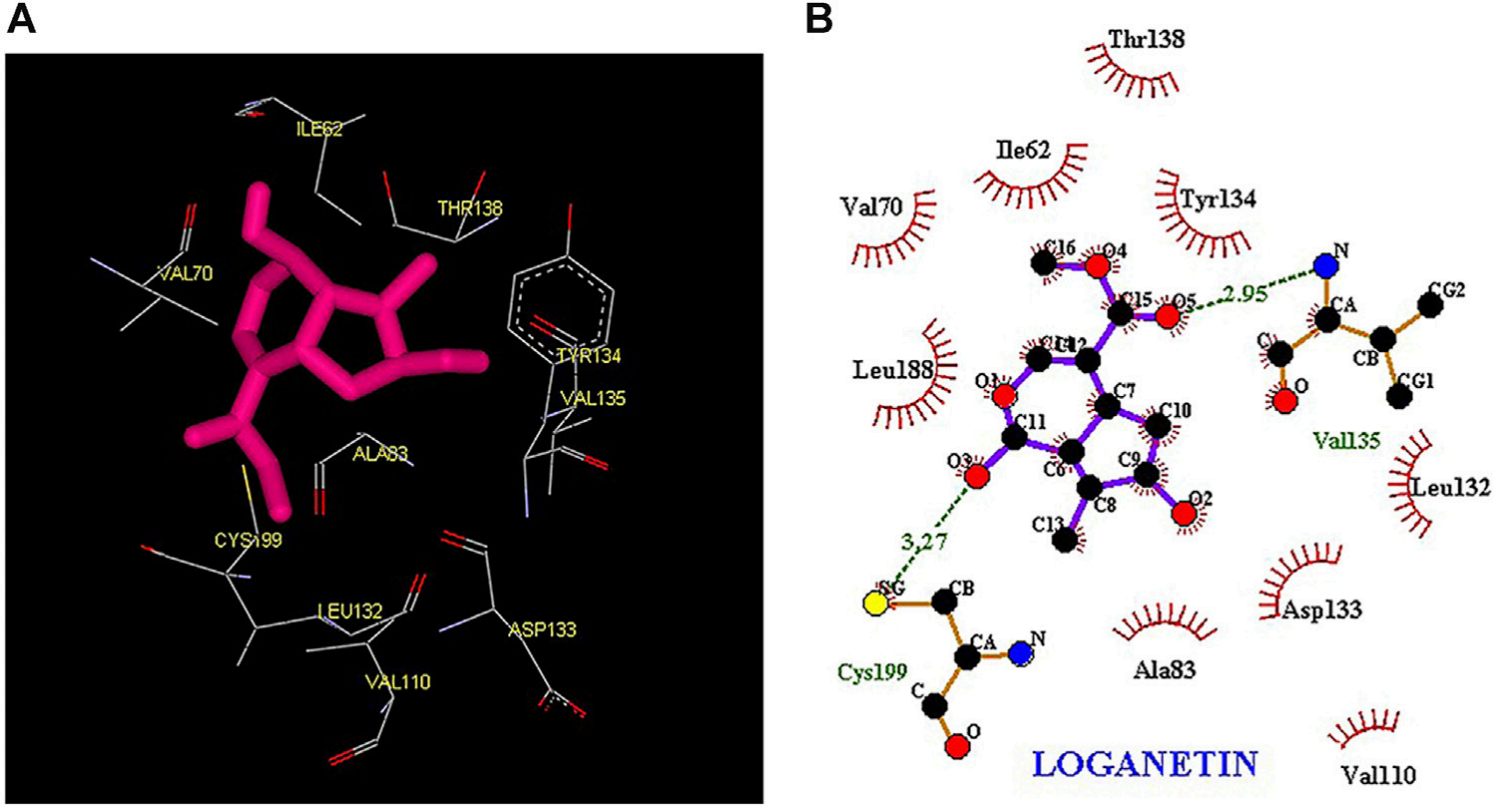
**(A)** Three-dimensional view of loganetin at the GSK_3β protein binding site. **(B)** Interactions of loganetin with amino acids of the GSK3_β protein binding site.

**FIGURE 4:**
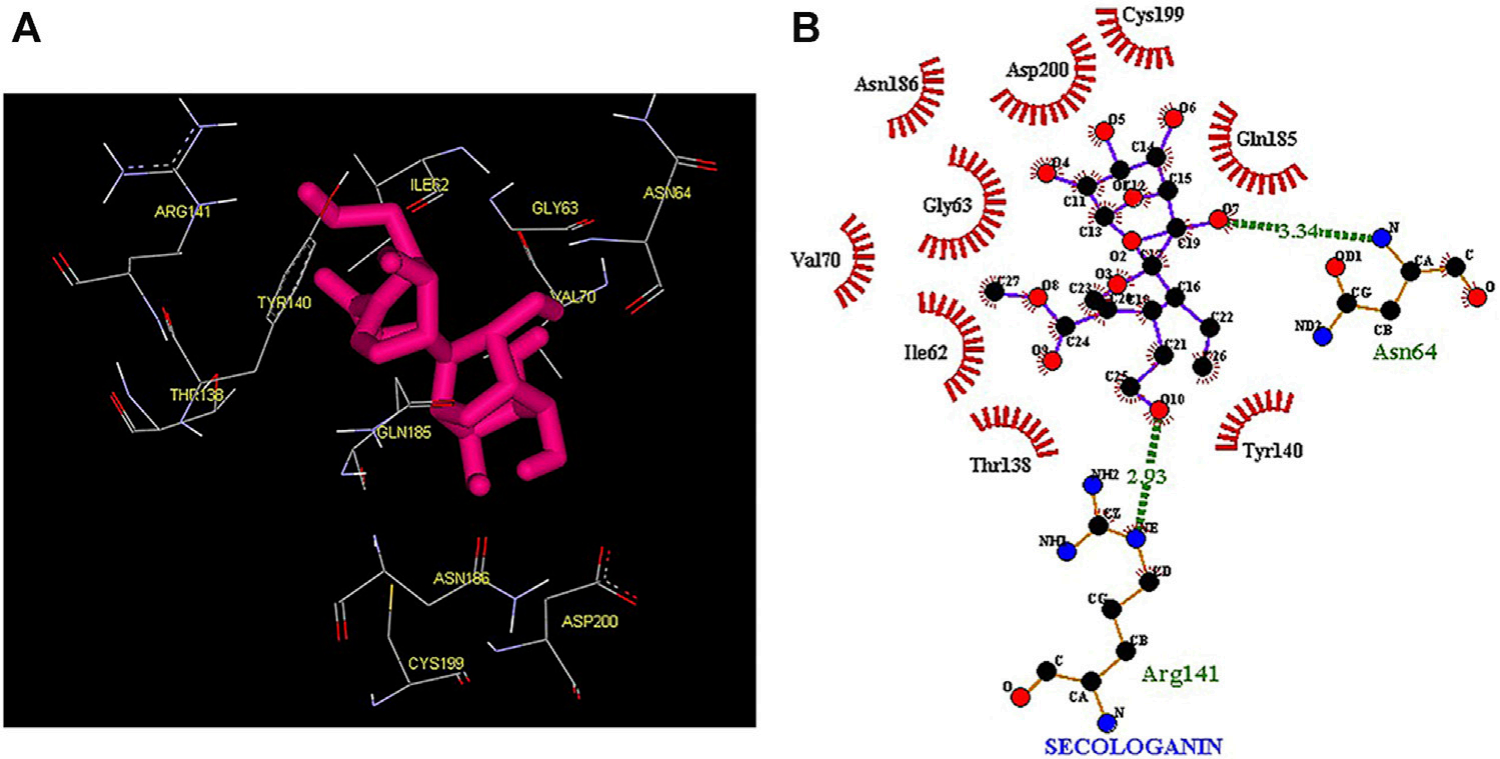
**(A)** Three-dimensional view of secologanin at the GSK_3β protein binding site. **(B)** Interactions of secologanin with amino acids of the GSK3_β protein binding site.

Thus, the docking method used in this study could effectively identify how the inhibitor binds to the enzyme. The average binding energy was, respectively, −7.15, −5.43, and −4.98 kcal/mol in the molecular docking of loganin, loganetin, and secologanin with GSK3-β protein. As can be seen, the binding energy of loganin was positive compared to that of BRW1383 but negative compared to that of other ligands. Docking results showed that loganin had a hydrogen bond with the amino acids VAL135 and ASP200 and a hydrophobic bond with ASP133. The details of the interaction between loganin and GSK3-β provided by Ligplot software are given in Figure 2B. As already stated, these three amino acids are the key amino acids in the regulation of GSK3-β protein (Figure 2A). However, BRW1383 has better binding energy vs. loganin, as shown in Table 2 which demonstrates that loganin interacts with all of the key amino acids in the active site, but the reference inhibitor interacts with just ASP133 and VAL135. As loganin is a natural compound, it can be a choice for inhibiting GSK3-β protein compared with BRW1383 which is a chemical compound. According to Figure 3, loganetin has established a hydrogen bond with VAL135 and a hydrophobic bond with ASP133. Additionally, secologanin has established two hydrogen bonds with ASN64 and ARG141 and several hydrophobic bonds outside the active site (Figure 4).

**TABLE 2:** Docking results of loganin, loganetin, secologanin, and BRW1383 with GSK3-β protein.

Ligand	Two-dimensional structure	Binding energy (Kcal.mol)	Hydrogen bonds	Bond length (A°)	Hydrophobic bonds
Loganin	fx1	−7.15	ASN64	3.24	Cys199
					Asp133
					Ala83
			Asp200	3.23	Val110
					Leu188
					Leu132
			Val135	3.23	Val70 Lys85
					Ile62 Gly63
Loganetin	fx2	−5.43	Val135	2.95	Asp133
					Leu132
					Ala83
			Cys199	3.27	Val110
					Tyr134
					Thr138
					Ile62
					Val70
					Leu188
Secologanin	fx3	−4.98	Asn64	3.34	Tyr140
					Gln185
					Cys199
					Asp200
			Arg141	2.93	Asn186
					Gly63
					Val70
					Ile62
					Thr138
BRW1383	fx4	−9.53	Val135	2.93	Ile62, Val70
				2.42	Ala83,
					Leu132
					Tyr134
			Asp133	2.90	Thr138,
					Arg141,
					Leu188
					Cys199

The results in Table 2 show that loganin binds more strongly to the active site of the GSK3-β protein compared to other ligands. To ensure the stability of the docked compound, the distance of hydrogen bonds between the compounds and the active site residues was investigated. According to Jeffrey’s study (Jeffrey 1997), the acceptable hydrogen bond distance between the donor and acceptor should be from 2.7 to 3.3 A°. The 2.2–2.5 Å distance is considered strong and covalent, 2.5–3.2 Å is considered medium and electrostatic bonds, and 3.2–4 Å is considered weak electrostatic bonds. Moreover, Ippolito and colleagues (1990) and Raschka and colleagues (2018) have revealed that the average distance between the protein and ligand from 2.4 to 3.5 Å could be considered hydrogen bonding.

Thus, the hydrogen bond interaction of our ligands with the protein was considered the medium hydrogen bond type. Therefore, in this study, the moderate hydrogen bond seems to be enough to inhibit the GSK3-β protein.

### Molecular Dynamic Simulation

Although the docking results provide good information on the quality and interactions of the ligand and protein, they cannot predict how the protein conformations will change after ligand binding. Thus, to examine the changes and dynamics of the protein in interaction with three selected ligands, simulations were performed throughout for 100 ns

The before and after MD simulation of protein–ligand interaction residues are compared in Table 3 (Saddala and Adi 2018).

**TABLE 3:** The before and after simulation of protein–ligand interaction residues.

Compound	Active site amino acids before simulation	Active site amino acids after simulation
**Loganin**	ASP133, VAL135, ASP200, TYR134	VAL135,ASP133,TYR134
**Loganetin**	VAL135,ASP133,TYR134	VAL135,ASP133, TYR134
**Secologanin**	ASP200	ASP200

**FIGURE 5:**
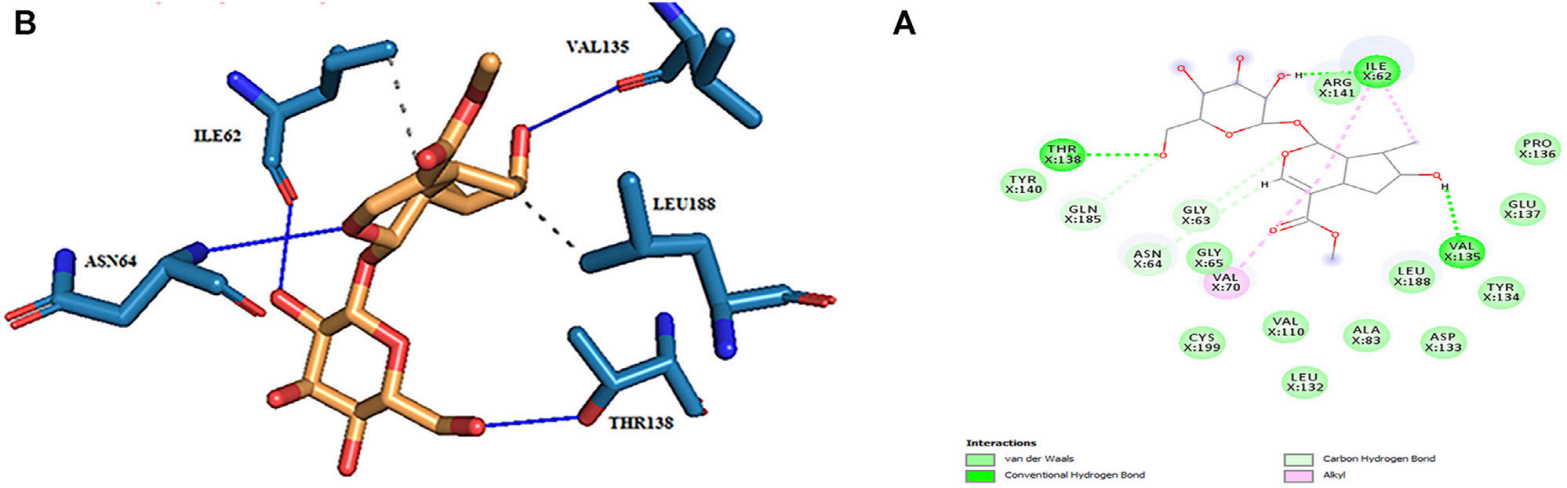
**(A)** 2d and **(B)** 3d view of the GSK_3β_loganin complex after MD simulation.

**FIGURE 6:**
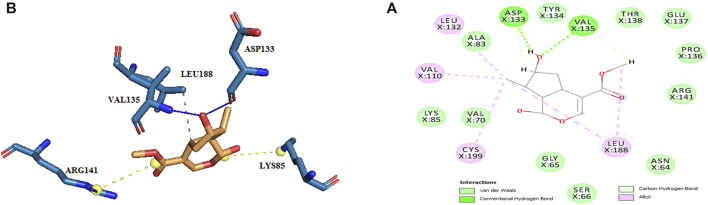
**(A)** 2d and **(B)** 3d view of the GSK_3β_loganetin complex after MD simulation.

**FIGURE 7:**
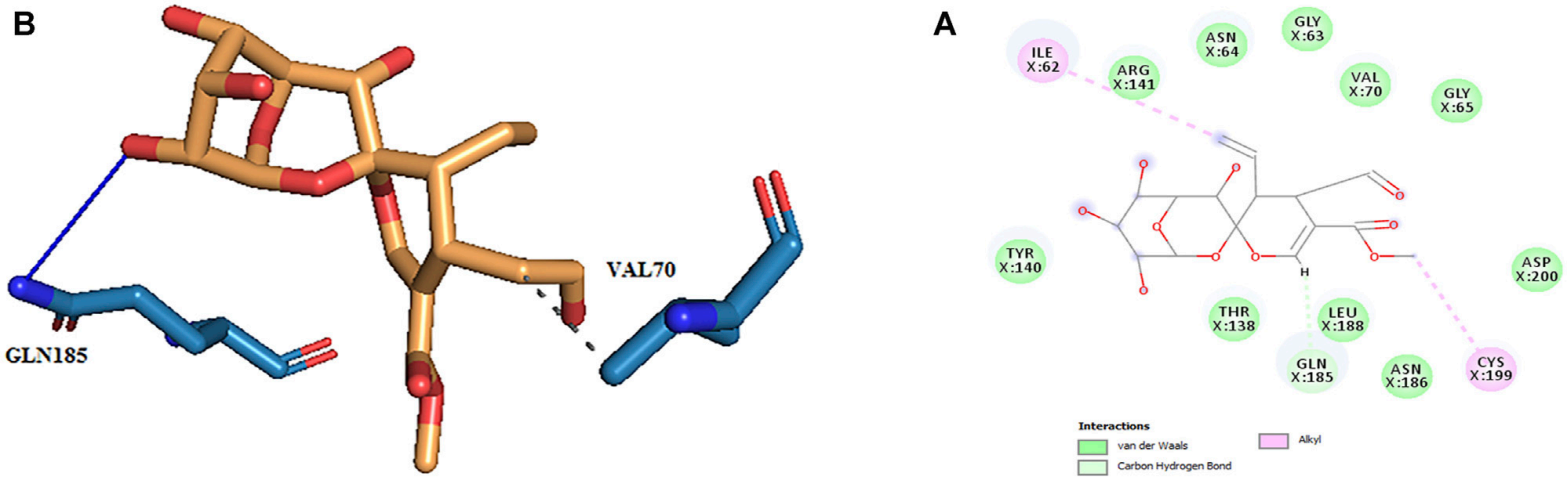
**(A)** 2d and **(B)** 3d view of the GSK_3β_secologanin complex after MD simulation.

The active site amino acids of all compounds in the molecular docking were almost similar to the active site residues in the MD simulation. Thus, these compounds have stayed stable in the active site after simulation and did not change the protein structure. Although, secologanin before and after simulation could not interact with active site residues well and just could interact with ASP200. Consequently, secologanin was not considered a GSK3-β inhibitor. Also, we have prepared 3d and 2d demonstrations of all compounds after MD simulation as shown in Figures 5–7. As can be seen, the most important functional groups of loganin and loganetin that participate in hydrogen bond interactions with active site residues are related to the sugar part of these iridoid glycosides.

In molecular docking, only the ligand has a flexible state. However, both the protein and ligand were considered flexible in MD simulation. Therefore, during MD simulation, we could evaluate all observed interactions in molecular docking and find potential and new effects regarding the conformational changes of the ligand and the enzyme-binding site. As shown in Table 4, in hydrogen bond examinations of the GSK3-β_loganin complex, the most stable interactions have been established between O val135 and H20 atoms with 97% occupancy and HG1 THR138 with O4 loganin with 94% occupancy. Here, val135 is the active site amino acid and is involved in the regulation of GSK3-β protein. Among the hydrogen bonds that loganin has established with the protein, only the hydrogen bond with val135 is identical to the docking results. Although the protein is considered rigid in docking, in MD simulation, both the protein and ligand are considered flexible; because of that, MD results are more reliable. It is also noteworthy that only two hydrogen bonds were predicted for secologanin in docking, but several hydrogen bonds have been predicted for MD. As Table 4 shows, the occupancy listed for each of the secologanin hydrogen bonds is nonsignificant, and none of these bonds are established in the protein active site.

**TABLE 4:** Analysis of intermolecular hydrogen bonds.

Ligand	Donor–acceptor	Occupancy (%)
Loganin	383LIG (H20)–135VAL(O)	80.1
	383LIG (H15)–62 ILE (O)	22.4
	138THR (HG1)–383LIG (O4)	94.5
	64ASN(HN)–383LIG (O8)	42.3
Loganetin	383LIG (H17)–133ASP(O)	84
	135VAL(HN)–383LIG (O2)	54.7
Secologanin	186ASN(D21)–383LIG (O4)	13.2
	183LYS(HZ1)–383LIG (O6)	15
	183LYS(HZ1)–383LIG (O4)	22.4
	66SER(HN)–383LIG (O5)	13
	64ASN(HN)–383LIG (O5)	14.9

Loganetin has two other hydrogen bonds, where its H17 atom is bonded to oxygen ASP133, and its O2 atom is bonded to HN VAL135, whereas these two amino acids are of the residues of the active site of proteins; however, the occupancy of the loganin hydrogen bonds is greater. Thus, loganin binds more strongly to the protein compared to the other two ligands.

### Root Mean Square Deviation

The first critical analysis of MD simulation is the RMSD, which shows the stability and structural changes during the simulation. RMSD is a parameter that shows the deviation of the particle position from the original structure at any point in time (Czelen 2017; Sargsyan et al., 2017).

The lower RMSD value indicates less fluctuation during the simulation, suggesting that the stability of the protein is high (Elangovan et al., 2020). Therefore, the system will be balanced. Figure 6 presents the variation in RMSD values of GSK3-β protein comparing with the three complexes. The RMSD value revealed that all pathways reached equilibrium after 10 ns Thus, RMSF, Rg, SASA, and hydrogen bonds were analyzed at an equilibrium state.

**FIGURE 8:**
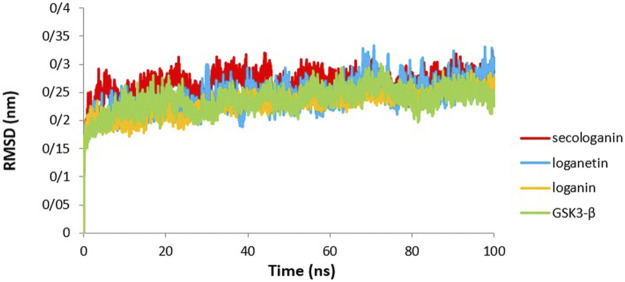
RMSD analysis GSK3-β protein (green), loganin-GSK3-β complex (yellow), loganetin-GSK3-β complex (blue), and secologanin-GSK3-β complex (red).

As Figure 8 shows for the GSK3-β protein (green diagram), the mean RMSD has remained at about 0.19 nm with minimal fluctuation. Consequently, the protein does not undergo severe deformation and denaturation while simulating.

As can be seen from the GSK3-β_secologanin complex, the RMSD has higher values and fluctuations (0.23 nm) relative to the free protein and compared to loganin and loganetin about 0.19 and 0.21, respectively. Thus, the protein–secologanin complex is more unstable than other structures. Here, the protein–loganin RMSD compared to GSK3-β protein is more stable than the other two complexes. Also, the enzyme structure has not changed significantly in the presence of loganin, and the system is stable during the simulation.

### Root Mean Square Fluctuation

Another used parameter to measure stability and flexibility is the RMSF during 90 ns. The RMSF estimates the average deviation of a particle (like protein residues) from a reference position (typically the average particle position) over time (Reddy et al., 2015).

Therefore, RMSF analyzes those parts of the structure with the most and least fluctuations from their dependent structure, and this parameter describes how ligand binding can result in conformational changes at the residual level (Elangovan et al., 2020; Saravanan et al., 2020).

Overall, the peaks seen in the RMSF diagram showed the most unstable target protein residues.

**FIGURE 9:**
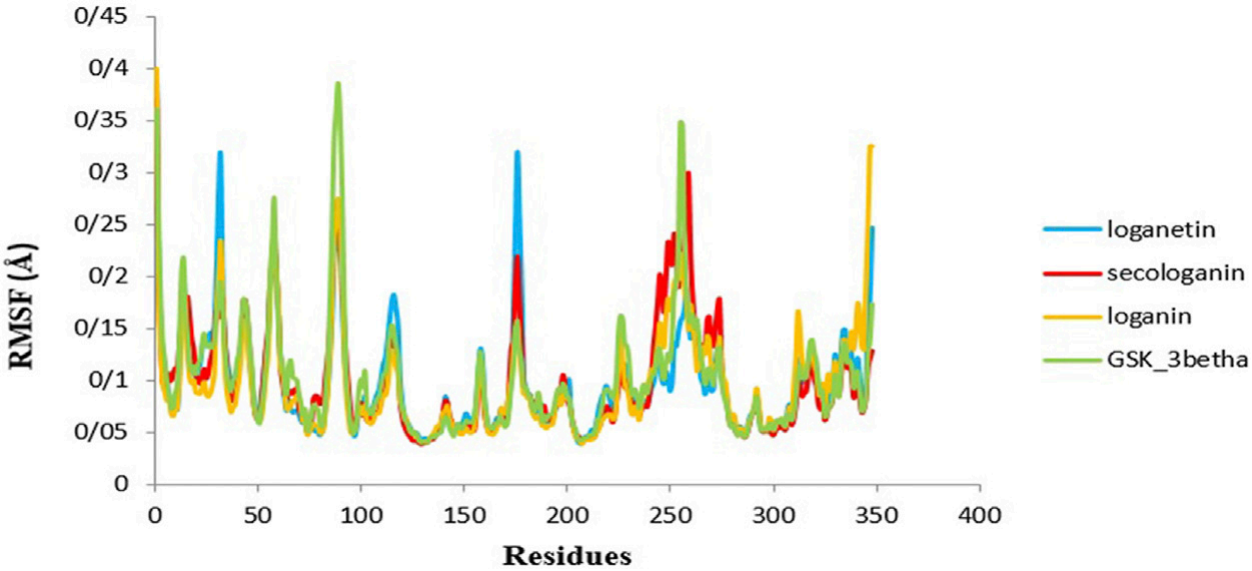
RMSF analysis GSK3-β protein (green), loganin-GSK3-β complex (yellow), loganetin-GSK3-β complex (blue), and secologanin-GSK3-β complex (red).

Figure 9 illustrates the high residue fluctuations at the start and end of the RMSF chart.

Moreover, from Figure 9, loganin has less fluctuation compare with loganetin and secologanin, and it has the most adaption with the RMSF GSK3-β protein diagram.

Moreover, amino acids' RMSF of the active site in each of the three ligands reveals that binding of the ligand to the protein in the active site does not cause the fluctuation of residues, and the structure remains stable. The mean RMSF values for GSK3-β protein, loganin, loganetin, and secologanin were 2.16, 2.16, 2.17, and 2.15, respectively. These values suggest that ligand binding does not change the original conformation of the residues. Also, it is inferred that the compounds do not fluctuate much, they seem consistent with protein fluctuations, and the complexes seem stable.

### Radius of Gyration

The gyration radius is a variable that is studied to examine the compression changes during MD simulation. Protein compression during interactions with the ligand is affected by protein chains, in which the flexibility between the ligand and the protein depends on it (Shukla et al., 2019; Ghosh et al., 2020).

**FIGURE 10:**
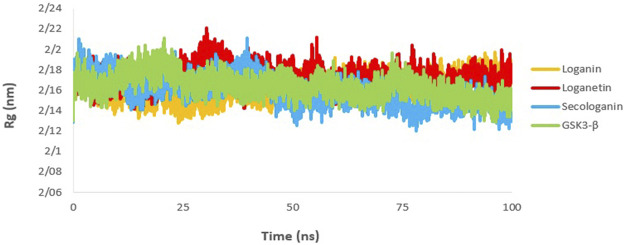
Rg analysis GSK3-β protein (green), loganin-GSK3-β complex (yellow), loganetin-GSK3-β complex (blue), and secologanin-GSK3-β complex (red).

The calculated Rg value for the three complexes (Figure 10) was 2.16, which is associated with the protein size as well. The Rg of GSK3-β protein was 2.16, indicating that GSK3-β protein remains compressed during MD simulation and binding to all three ligands.

### Solvent-Accessible Surface Area

The SASA value is used to analyze the magnitude and significance of ligand binding to the receptor and the changes in protein conformation due to ligand binding (Shukla et al., 2019; Elangovan et al., 2020).

**FIGURE 11:**
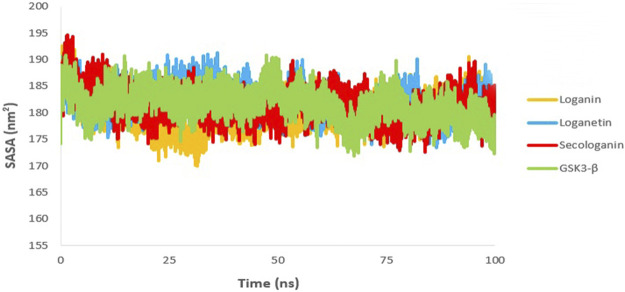
SASA analysis GSK3-β protein (green), loganin-GSK3-β complex (yellow), loganetin-GSK3-β complex (blue), and secologanin-GSK3-β complex (red).

As shown in Figure 11, all complexes show a similar magnitude of protein conformation that interacts with the ligand compared to the GSK3-β protein.

On the other hand, SASA changes are similar to the changes in the Rg diagram, confirming the accuracy of the molecular dynamic simulations. Thus, one can infer that all protein–ligand complexes are stable in the SASA analysis.

### The Analysis of Hydrogen Bonds

**FIGURE 12:**
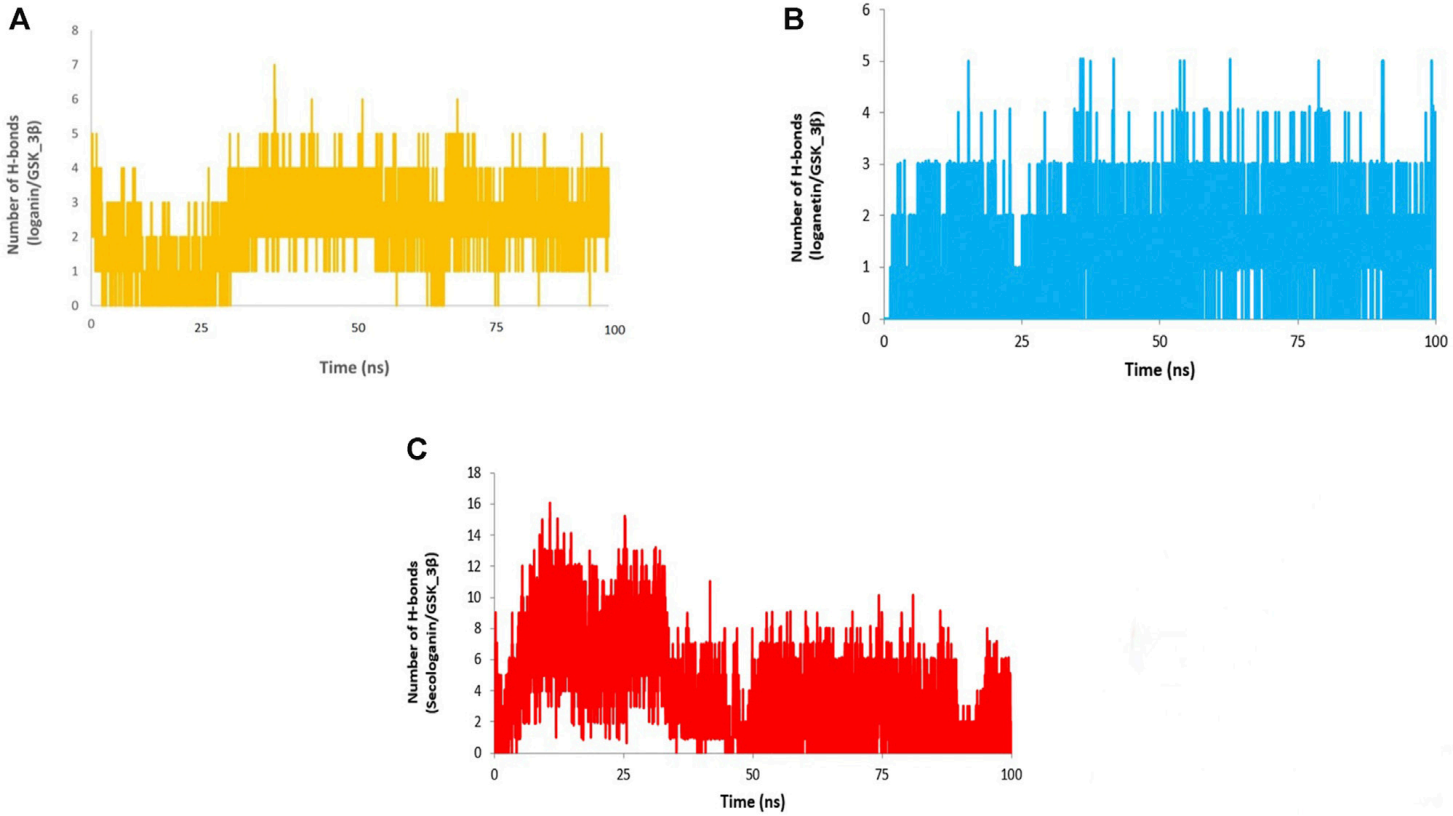
H-bond analysis **(A)** loganin, **(B)** loganetin, and **(C)** secologanin.

Different interactions like hydrogen bonds, hydrophobic interactions, and ionic interactions stabilize the protein–ligand complex. Between them, the hydrogen bonds are more important and specific transient interactions than others for protein–ligand stabilization (Shukla et al., 2019). The number of hydrogen bonds was calculated for the last 90 ns trajectory. The number of hydrogen bonds vs. time is revealed for each complex in Figure 12. The average number of hydrogen bonds for loganin_GSK3-β (Figure 12A) and loganetin_GSK3-β was (Figure 12B) 3 and 2, respectively. But the hydrogen bonds of secologanin_GSK3-β are variable.

As can be seen, a partial change in the hydrogen bond formation between the ligand and protein for loganin_GSK3-β and loganetin_GSK3-β was observed. So, these complexes were stable for most parts of the simulation trajectory compared to secologanin_GSK3-β. These results are approximately similar to the results that are presented in Table 4.

### Binding Free Energy Calculation

The binding free energy is a critical step of the in silico drug design approach which determines the binding affinity of inhibitors to the receptor. The binding free energy was calculated for the ligand receptor of loganin, loganetin, and secologanin complexes using the MM/PBSA method. The nonpolar and polar solvation energy was analyzed in the electrostatic interaction, van der Waals energy, and SASA energy. The major desirable portions of the stereoisomer binding were the van der Waals and electrostatic energies for all complexes. Besides, the terms polar solvation free energy and SASA energy are unfavorable for binding in three complexes. The total free binding energy (ΔG_Binding_) was calculated for each compound in Table 5.

**TABLE 5:** Table represents the Van der Waals, electrostatic, polar solvation, SASA, and binding energy in kJ.mol_1 for secologanin, loganetin, and loganin.

Compound	van der Waals energy	Electrostatic energy	Polar solvation energy	SASA energy	Binding energy
**Secologanin**	−150.661 ± 11.942	−21.726 ± 12.326	187.567 ± 826.391	−17.050 ± 1.152	−1.870 ± 827.549
**Loganetin**	−105.747 ± 8.947	−30.152 ± 8.113	95.579 ± 16.494	−12.559 ± 0.718	−52.878 ± 11.941
**Loganin**	−157.429 ± 12.579	−44.508 ± 12.625	168.159 ± 15.026	−18.644 ± 0.932	−52.421 ± 14.661

Loganin and loganetin have an almost similar amounts of binding energy, respectively, −52.421 and −52.878 KJ/mol, but they are much higher than that of secologanin (−1.870 KJ/mol). We have concluded that loganin and loganetin are more energetically favorable than secologanin, and these results are adopted with docking conclusions.

### Drug Likeness Prediction and Toxicity

Pharmacokinetic properties and toxicities were predicted for each compound used by the OSIRIS property explorer server, and the results are revealed within Table 6. The mutagenicity, tumorigenicity, irritation force, and hazard of the reproductive force were predicted for toxicity verification.

**TABLE 6:** Drug likeness prediction and toxicity of loganin, loganetin, and secologanin.

Properties	Loganin	Loganetin	Secologanin
**Mutagenic**	NO	NO	NO
**Tumorigenic**	NO	NO	NO
**Irritant**	NO	NO	High risk fragment indicating irritating effects
**Reproductive effective**	NO	NO	NO
**C Log p**	−1.97	−0.13	−1.85
**Solubility**	−1.16	−1.46	−1.23
**MW**	390	228	388
**TPSA**	155.1	76.99	151.9
**Drug likeness**	−3.92	−0.11	−10.16
**Drug score**	0.45	0.71	0.27

The higher cLog P (logarithm of compounds' partition coefficient between water and n-octanol) value indicates lower permeation and absorption due to lower hydrophilicity. The solubility and molecular weight (MW) affect the absorption rate; thus, high solubility and lower MW increase absorption. The topological polar surface area (TPSA) reveals the surface of polar atoms in the compounds. An increased TPSA value is relevant to the least permeability of the membrane. So, the compounds with a larger TPSA value will be a better substrate for p-glycoprotein which is amenable for the efflux of a drug from the cell, and thus, the diminished TPSA was useful for the drug-like property. Some investigations also predicted that a molecule with a better CNS penetration should have a lesser value of the TPSA (Reddy et al., 2015; Srivastava et al., 2020).

Furthermore, the drug score mixes drug likeness, clog P, TPSA, MW, and toxicity risk parameters to reveal a compound as a drug candidate. Based on Table 6, loganetin has a higher score (0.71) than loganin (0.45) and secologanin (0.27) because loganin has a lower TPSA, MW, and cLog P and higher solubility than other compounds.

## Conclusion

This study aimed at finding potential and available inhibitors for GSK3-β protein using computational drug design methods like molecular docking and MD simulation.

We performed molecular analysis of the iridoid glycosides in the Common snowberry plant, including loganin, secologanin, and loganetin, which have beneficial effects on improving memory.

Molecular docking analysis revealed that loganin and loganetin bind exactly to the ATP-binding site. Loganin established hydrogen bonding with VAL135 and ASP200 and hydrophobic bonding with ASP133, and loganetin established hydrogen bonding with VAL135 and hydrophobic bonding with ASP133, but the binding affinity of loganin was more than that of loganetin.

RMSD, RMSF, Rg, SASA, hydrogen bond analyses, and MMPBSA were carried out in the molecular dynamic simulation. The results of the RMSD value indicated that the loganin–GSK3 complex has more stability than the other two complexes.

The RMSF diagram indicated that ligand binding does not increase fluctuations, and the complexes stay stable following the binding.

The SASA analysis was equal with variations of the Rg value, and equality of the Rg value of the GSK3-β protein with complexes indicates that the structure remains compact during MD simulation.

Loganin and loganetin have an almost similar amount of binding energy, and loganetin has a better drug score than others.

Consequently, both loganin and loganetin may effectively inhibit GSK3-β because these compounds established strong hydrogen bonds with the active site residues before and after MD simulation. Although the binding affinity of loganin and loganetin was slightly less than that of the BRW1383 inhibitor in molecular docking, they can be considered to prevent tauopathy since those are available and nontoxic herbal compounds. Nonetheless, this study requires more examination, and it is better to carry out laboratory studies along with molecular studies to prove the effects of iridoid glycosides on AD prevention and treatment.

## Data Availability

The original contributions presented in the study are included in the article/Supplementary Materials, further inquiries can be directed to the corresponding author.

## References

[B1] Allard J. (2004). "From Genetics to Therapeutics: the Wnt Pathway and Osteoporosis." 1r0e PDB entry.

[B2] Arnost M. Pierce A. Haar E. t. Lauffer D. Madden J. Tanner K. (2010). 3-Aryl-4-(arylhydrazono)-1H-pyrazol-5-ones: Highly Ligand Efficient and Potent Inhibitors of GSK3β. Bioorg. Med. Chem. Lett. 20 (5), 1661–1664. 10.1016/j.bmcl.2010.01.072 20138514

[B3] Aronov A. M. Tang Q. Martinez-Botella G. Bemis G. W. Cao J. Chen G. (2009). Structure-guided Design of Potent and Selective Pyrimidylpyrrole Inhibitors of Extracellular Signal-Regulated Kinase (ERK) Using Conformational Control. J. Med. Chem. 52 (20), 6362–6368. 10.1021/jm900630q 19827834

[B4] Association A. s. (2019). 2019 Alzheimer's Disease Facts and Figures. Alzheimer's Demen. 15 (3), 321–387. 10.1016/j.jalz.2019.01.010

[B5] Bagyinszky E. Youn Y. C. An S. Kim S. (2014). The Genetics of Alzheimer's Disease. Cia 9, 535. 10.2147/cia.s51571 PMC3979693 24729694

[B6] Bertrand J. A. Thieffine S. Vulpetti A. Cristiani C. Valsasina B. Knapp S. (2003). Structural Characterization of the GSK-3β Active Site Using Selective and Non-selective ATP-Mimetic Inhibitors. J. Mol. Biol. 333 (2), 393–407. 10.1016/j.jmb.2003.08.031 14529625

[B7] Bhat R. Xue Y. Berg S. Hellberg S. Ormö M. Nilsson Y. (2003). Structural Insights and Biological Effects of Glycogen Synthase Kinase 3-specific Inhibitor AR-A014418. J. Biol. Chem. 278 (46), 45937–45945. 10.1074/jbc.m306268200 12928438

[B8] Brookmeyer R. Johnson E. Ziegler-Graham K. Arrighi H. M. (2007). Forecasting the Global burden of Alzheimer's Disease. Alzheimer's Demen. 3 (3), 186–191. 10.1016/j.jalz.2007.04.381 19595937

[B9] Coffman K. Brodney M. Cook J. Lanyon L. Pandit J. Sakya S. (2011). 6-Amino-4-(pyrimidin-4-yl)pyridones: Novel Glycogen Synthase Kinase-3β Inhibitors. Bioorg. Med. Chem. Lett. 21 (5), 1429–1433. 10.1016/j.bmcl.2011.01.017 21295469

[B10] Czeleń P. (2017). Inhibition Mechanism of CDK-2 and GSK-3β by a Sulfamoylphenyl Derivative of Indoline—A Molecular Dynamics Study. J. Mol. Model. 23 (8), 1–11. 28726150 10.1007/s00894-017-3395-8 PMC5517586

[B11] Davies M. P. (2019). Silico Screening and *in Vitro* Evaluation of GSK-3β Type I and II Inhibitors: Potential Treatment for Alzheimer’s Disease? University of Central Lancashire.

[B12] De Strooper B. Karran E. (2016). The Cellular Phase of Alzheimer's Disease. Cell 164 (4), 603–615. 10.1016/j.cell.2015.12.056 26871627

[B13] Dinda B. Debnath S. Harigaya Y. (2007). Naturally Occurring Secoiridoids and Bioactivity of Naturally Occurring Iridoids and Secoiridoids. A Review, Part 2. Chem. Pharm. Bull. 55 (5), 689–728. 10.1248/cpb.55.689 17473457

[B14] Dinda B. Dinda M. Kulsi G. Chakraborty A. Dinda S. (2019). Therapeutic Potentials of Plant Iridoids in Alzheimer's and Parkinson's Diseases: A Review. Eur. J. Med. Chem. 169, 185–199. 10.1016/j.ejmech.2019.03.009 30877973

[B15] Eftekharzadeh B. Daigle J. G. Kapinos L. E. Coyne A. Schiantarelli J. Carlomagno Y. (2018). Tau Protein Disrupts Nucleocytoplasmic Transport in Alzheimer's Disease. Neuron 99 (5), 925–940. 10.1016/j.neuron.2018.07.039 30189209 PMC6240334

[B16] Elangovan N. D. Dhanabalan A. K. Gunasekaran K. Kandimalla R. Sankarganesh D. (2020). Screening of Potential Drug for Alzheimer's Disease: a Computational Study with GSK-3 β Inhibition through Virtual Screening, Docking, and Molecular Dynamics Simulation. J. Biomol. Struct. Dyn., 1–15. 10.1080/07391102.2020.1805362 32779973

[B17] Forli S. Olson A. J. (2012). A Force Field with Discrete Displaceable Waters and Desolvation Entropy for Hydrated Ligand Docking. J. Med. Chem. 55 (2), 623–638. 10.1021/jm2005145 22148468 PMC3319101

[B18] Gharaghani S. Khayamian T. Ebrahimi M. (2013). Molecular Dynamics Simulation Study and Molecular Docking Descriptors in Structure-Based QSAR on Acetylcholinesterase (AChE) Inhibitors. SAR QSAR Environ. Res. 24 (9), 773–794. 10.1080/1062936x.2013.792877 23863115

[B19] Ghosh R. Chakraborty A. Biswas A. Chowdhuri S. (2020). Identification of Polyphenols from Broussonetia Papyrifera as SARS CoV-2 Main Protease Inhibitors Using In Silico Docking and Molecular Dynamics Simulation Approaches. J. Biomol. Struct. Dyn., 1–14. 10.1080/07391102.2020.1802347 PMC7484588 32762411

[B20] Gilbert O. L. (1995). Symphoricarpos Albus (L.) S. F. Blake (S. Rivularis Suksd., S. Racemosus Michaux). J. Ecol. 83 (1), 159–166. 10.2307/2261160

[B21] Gong C.-X. Iqbal K. (2008). Hyperphosphorylation of Microtubule-Associated Protein Tau: a Promising Therapeutic Target for Alzheimer Disease. Cmc 15 (23), 2321–2328. 10.2174/092986708785909111 PMC2656563 18855662

[B22] Gyurak A. Goodkind M. S. Kramer J. H. Miller B. L. Levenson R. W. (2012). Executive Functions and the Down-Regulation and Up-Regulation of Emotion. Cogn. Emot. 26 (1), 103–118. 10.1080/02699931.2011.557291 21432634 PMC3155745

[B23] Hall A. Pekkala T. Polvikoski T. van Gils M. Kivipelto M. Lötjönen J. (2019). Prediction Models for Dementia and Neuropathology in the Oldest Old: the Vantaa 85+ Cohort Study. Alzheimers Res. Ther. 11 (1), 11–12. 10.1186/s13195-018-0450-3 30670070 PMC6343349

[B24] Ippolito J. A. Alexander R. S. Christianson D. W. (1990). Hydrogen Bond Stereochemistry in Protein Structure and Function. J. Mol. Biol. 215 (3), 457–471. 10.1016/s0022-2836(05)80364-x 2231715

[B25] Jeffrey G. (1997). An Introduction to Hydrogen Bonding. New York: Oxford University PressGoogle Scholar There is no corresponding record for this reference, 220–225.

[B26] Jouanne M. Rault S. Voisin-Chiret A.-S. (2017). Tau Protein Aggregation in Alzheimer's Disease: an Attractive Target for the Development of Novel Therapeutic Agents. Eur. J. Med. Chem. 139, 153–167. 10.1016/j.ejmech.2017.07.070 28800454

[B27] Kametani F. Hasegawa M. (2018). Reconsideration of Amyloid Hypothesis and Tau Hypothesis in Alzheimer's Disease. Front. Neurosci. 12, 25. 10.3389/fnins.2018.00025 29440986 PMC5797629

[B28] Kaplan W. Littlejohn T. G. (2001). Swiss-PDB Viewer (Deep View). Brief. Bioinformatics 2 (2), 195–197. 10.1093/bib/2.2.195 11465736

[B29] Kolarova M. García-Sierra F. Bartos A. Ricny J. Ripova D. (2012). Structure and Pathology of Tau Protein in Alzheimer Disease. Int. J. Alzheimers Dis. 2012, 731526. 10.1155/2012/731526 22690349 PMC3368361

[B30] Laskowski R. A. Swindells M. B. (2011). LigPlot+: Multiple Ligand–Protein Interaction Diagrams for Drug Discovery. European Bioinformatics Institute. 10.1021/ci200227u 21919503

[B31] Lauretti E. Dincer O. Praticò D. (2020). Glycogen Synthase Kinase-3 Signaling in Alzheimer's Disease. Biochim. Biophys. Acta (Bba) - Mol. Cel Res. 1867 (5), 118664. 10.1016/j.bbamcr.2020.118664 PMC7047718 32006534

[B32] Lee S. J. Chung Y. H. Joo K. M. Lim H. C. Jeon G. S. Kim D. (2006). Age-related Changes in Glycogen Synthase Kinase 3β (GSK3β) Immunoreactivity in the central Nervous System of Rats. Neurosci. Lett. 409 (2), 134–139. 10.1016/j.neulet.2006.09.026 17046157

[B33] Lin R. Jones N. C. Kwan P. (2020). Unravelling the Role of Glycogen Synthase Kinase-3 in Alzheimer's Disease-Related Epileptic Seizures. Ijms 21 (10), 3676. 10.3390/ijms21103676 PMC7279454 32456185

[B34] Llorens-Marítin M. Jurado J. Hernández F. Avila J. (2014). GSK-3β, a Pivotal Kinase in Alzheimer Disease. Front. Mol. Neurosci. 7, 46. 24904272 10.3389/fnmol.2014.00046 PMC4033045

[B35] Ma D. Zhu Y. Li Y. Yang C. Zhang L. Li Y. (2016). Beneficial Effects of Cornel Iridoid Glycoside on Behavioral Impairment and Senescence Status in SAMP8 Mice at Different Ages. Behav. Brain Res. 312, 20–29. 10.1016/j.bbr.2016.06.008 27283974

[B36] Makarevich I. F. Kovalenko S. N. Gusarova T. D. Gubin Y. I. (2009). Iridoids from Symphoricarpos Albus. Chem. Nat. Compd. 45 (1), 40–44. 10.1007/s10600-009-9257-6

[B37] Mandelkow E. von Bergen M. Biernat J. Mandelkow E.-M. (2007). Structural Principles of Tau and the Paired Helical Filaments of Alzheimer?s Disease. Brain Pathol. 17 (1), 83–90. 10.1111/j.1750-3639.2007.00053.x 17493042 PMC8095506

[B38] Martin L. Latypova X. Wilson C. M. Magnaudeix A. Perrin M.-L. Yardin C. (2013). Tau Protein Kinases: Involvement in Alzheimer's Disease. Ageing Res. Rev. 12 (1), 289–309. 10.1016/j.arr.2012.06.003 22742992

[B39] Meijer L. Skaltsounis A.-L. Magiatis P. Polychronopoulos P. Knockaert M. Leost M. (2003). GSK-3-selective Inhibitors Derived from Tyrian Purple Indirubins. Chem. Biol. 10 (12), 1255–1266. 10.1016/j.chembiol.2003.11.010 14700633

[B40] Mendelsohn L. D. (2004). ChemDraw 8 Ultra, Windows and Macintosh Versions. J. Chem. Inf. Comput. Sci. 44, 2225–2226. 10.1021/ci040123t

[B41] Morris G. M. Goodsell D. S. Halliday R. S. Huey R. Hart W. E. Belew R. K. (1998). Automated Docking Using a Lamarckian Genetic Algorithm and an Empirical Binding Free Energy Function. J. Comput. Chem. 19 (14), 1639–1662. 10.1002/(sici)1096-987x(19981115)19:14<1639:aid-jcc10>3.0.co;2-b

[B42] Padhi A. K. Seal A. Khan J. M. Ahamed M. Tripathi T. (2021). Unraveling the Mechanism of Arbidol Binding and Inhibition of SARS-CoV-2: Insights from Atomistic Simulations. Eur. J. Pharmacol. 894, 173836. 10.1016/j.ejphar.2020.173836 33387467 PMC7773528

[B43] Pandey M. K. DeGrado T. R. (2016). Glycogen Synthase Kinase-3 (GSK-3)-Targeted Therapy and Imaging. Theranostics 6 (4), 571–593. 10.7150/thno.14334 26941849 PMC4775866

[B44] Raschka S. Wolf A. J. Bemister-Buffington J. Kuhn L. A. (2018). Protein-ligand Interfaces Are Polarized: Discovery of a strong Trend for Intermolecular Hydrogen Bonds to Favor Donors on the Protein Side with Implications for Predicting and Designing Ligand Complexes. J. Comput. Aided Mol. Des. 32 (4), 511–528. 10.1007/s10822-018-0105-2 29435780

[B45] Reddy S. V. G. Reddy K. T. Kumari V. V. Basha S. H. (2015). Molecular Docking and Dynamic Simulation Studies Evidenced Plausible Immunotherapeutic Anticancer Property by Withaferin A Targeting Indoleamine 2,3-dioxygenase. J. Biomol. Struct. Dyn. 33 (12), 2695–2709. 10.1080/07391102.2015.1004834 25671592

[B46] Saddala M. S. Adi P. J. (2018). Discovery of Small Molecules through Pharmacophore Modeling, Docking and Molecular Dynamics Simulation against Plasmodium Vivax Vivapain-3 (VP-3). Heliyon 4 (5), e00612. 10.1016/j.heliyon.2018.e00612 29756074 PMC5944417

[B47] Saitoh M. Kunitomo J. Kimura E. Hayase Y. Kobayashi H. Uchiyama N. (2009). Design, Synthesis and Structure-Activity Relationships of 1,3,4-oxadiazole Derivatives as Novel Inhibitors of Glycogen Synthase Kinase-3β. Bioorg. Med. Chem. 17 (5), 2017–2029. 10.1016/j.bmc.2009.01.019 19200745

[B48] Saitoh M. Kunitomo J. Kimura E. Iwashita H. Uno Y. Onishi T. (2009). 2-{3-[4-(Alkylsulfinyl)phenyl]-1-benzofuran-5-yl}-5-methyl-1,3,4-oxadiazole Derivatives as Novel Inhibitors of Glycogen Synthase Kinase-3β with Good Brain Permeability. J. Med. Chem. 52 (20), 6270–6286. 10.1021/jm900647e 19775160

[B49] Saravanan K. Hunday G. Kumaradhas P. (2020). Binding and Stability of Indirubin-3-Monoxime in the GSK3β Enzyme: a Molecular Dynamics Simulation and Binding Free Energy Study. J. Biomol. Struct. Dyn. 38 (4), 957–974. 10.1080/07391102.2019.1591301 30963817

[B50] Sargsyan K. Grauffel C. Lim C. (2017). How Molecular Size Impacts RMSD Applications in Molecular Dynamics Simulations. J. Chem. Theor. Comput. 13 (4), 1518–1524. 10.1021/acs.jctc.7b00028 28267328

[B51] Shahlaei M. Madadkar-Sobhani A. Fassihi A. Saghaie L. (2011). Exploring a Model of a Chemokine Receptor/ligand Complex in an Explicit Membrane Environment by Molecular Dynamics Simulation: the Human CCR1 Receptor. J. Chem. Inf. Model. 51 (10), 2717–2730. 10.1021/ci200261f 21910472

[B52] Shin D. Lee S.-C. Heo Y.-S. Lee W.-Y. Cho Y.-S. Kim Y. E. (2007). Design and Synthesis of 7-Hydroxy-1h-Benzoimidazole Derivatives as Novel Inhibitors of Glycogen Synthase Kinase-3β. Bioorg. Med. Chem. Lett. 17 (20), 5686–5689. 10.1016/j.bmcl.2007.07.056 17764934

[B53] Shukla R. Munjal N. S. Singh T. R. (2019). Identification of Novel Small Molecules against GSK3β for Alzheimer's Disease Using Chemoinformatics Approach. J. Mol. Graphics Model. 91, 91–104. 10.1016/j.jmgm.2019.06.008 31202091

[B54] Srivastava V. Yadav A. Sarkar P. (2020). Molecular Docking and ADMET Study of Bioactive Compounds of Glycyrrhiza Glabra against Main Protease of SARS-CoV2. Mater. Today Proc. 10.1016/j.matpr.2020.10.055 PMC7556787 33078096

[B55] ter Haar E. Coll J. T. Austen D. A. Hsiao H.-M. Swenson L. Jain J. (2001). Structure of GSK3β Reveals a Primed Phosphorylation Mechanism. Nat. Struct. Biol. 8 (7), 593–596. 10.1038/89624 11427888

[B56] Trott O. Olson A. J. (2010). AutoDock Vina: Improving the Speed and Accuracy of Docking with a New Scoring Function, Efficient Optimization, and Multithreading. J. Comput. Chem. 31 (2), 455–461. 10.1002/jcc.21334 19499576 PMC3041641

[B57] Lite V. (1998). Version 5.0. Accelrys Inc 9685.

[B58] Wang C. Gong X. Bo A. Zhang L. Zhang M. Zang E. (2020). Iridoids: Research Advances in Their Phytochemistry, Biological Activities, and Pharmacokinetics. Molecules 25 (2), 287. 10.3390/molecules25020287 PMC7024201 31936853

[B59] Zhang H.-C. Boñaga L. V. R. Ye H. Derian C. K. Damiano B. P. Maryanoff B. E. (2007). Novel Bis(indolyl)maleimide Pyridinophanes that Are Potent, Selective Inhibitors of Glycogen Synthase Kinase-3. Bioorg. Med. Chem. Lett. 17 (10), 2863–2868. 10.1016/j.bmcl.2007.02.059 17350261

[B60] Zhang Y. Huang N. Lu H. Huang J. Jin H. Shi J. (2020). Icariin Protects against Sodium Azide-Induced Neurotoxicity by Activating the PI3K/Akt/GSK-3β Signaling Pathway. PeerJ 8, e8955. 10.7717/peerj.8955 32341897 PMC7179568

